# Analysis of endothelial gene polymorphisms in Spanish patients with vascular dementia and Alzheimer´s disease

**DOI:** 10.1038/s41598-023-39576-7

**Published:** 2023-08-18

**Authors:** Raquel Manso-Calderón, Purificación Cacabelos-Pérez, M. Dolores Sevillano-García, M. Elisa Herrero-Prieto, Rogelio González-Sarmiento

**Affiliations:** 1 Department of Neurology, Complejo Asistencial Universitario de Salamanca (CAUSA), Paseo de San Vicente 58-182, 37007 Salamanca, Spain; 2 Division of Neurology, Department of Internal Medicine, Complejo Asistencial de Ávila, Ávila, Spain; 3 Instituto de Investigación Biomédica de Salamanca (IBSAL), University of Salamanca, Salamanca, Spain; 4 Department of Neurology, Hospital Clínico Universitario de Santiago (CHUS), A Coruña, Spain; 5 Division of Neurology, Department of Internal Medicine, Hospital El Bierzo de Ponferrada, León, Spain; 6 Molecular Medicine Unit, Department of Medicine, University of Salamanca, Salamanca, Spain

**Keywords:** Molecular biology, Neuroscience, Neurology, Pathogenesis, Medical research, Genetic association study, Alzheimer's disease

## Abstract

There is increasing evidence for the involvement of blood–brain barrier (BBB) in vascular dementia (VaD) and Alzheimer´s disease (AD) pathogenesis. However, the role of endothelial function-related genes in these disorders remains unclear. We evaluated the association of four single-nucleotide polymorphisms (*VEGF*, *VEGFR2 and NOS3*) with diagnosis and rate of cognitive decline in AD and VaD in a Spanish case–control cohort (150 VaD, 147 AD and 150 controls). Participants carrying -604AA genotype in *VEGFR2* (rs2071559) were less susceptible to VaD after multiple testing. Further analysis for VaD subtype revealed a significant difference between small-vessel VaD patients and controls, but not for large-vessel VaD patients. In addition, -2578A and -460C alleles in *VEGF* (rs699947 and rs833061) showed to decrease the risk of AD, whereas *NOS3* (rs1799983) influenced disease progression. Our study supports previous findings of a deleterious effect of VEGFR2 reduced expression on small-vessel disease, but not on large-vessel disease; as well as a detrimental effect of down-regulating VEGF and eNOS in AD, affecting vascular permeability and neuronal survival. These data highlight the relevance of endothelial function and, therefore, BBB in both VaD and AD.

## Introduction

Alzheimer’s disease (AD) is the most common cause of dementia, followed by vascular dementia (VaD). AD is characterized by extracellular deposits of amyloid-β peptide (Aβ), intracellular neurofibrillary tangles containing hyperphosphorylated tau protein and neuronal loss, whereas VaD is due to clinical stroke or subclinical vascular brain injury^1,2^. Beyond the monogenic forms, the majority of AD and VaD cases are sporadic disorders resulting from the interaction of multiple genetic and environmental factors. To date, apolipoprotein E (*APOE*)-ε4 is the only genetic factor consistently associated with both disorders^3,4^. In VaD, heterogeneity of the cerebrovascular mechanisms underlying this condition (e.g., cardioembolic, atherosclerotic, ischemic, haemorrhagic, etc.) creates challenges for research. In AD, genome-wide association studies (GWAS) have proposed several new susceptibility genes, but these variants only suppose a modest level of risk and its mechanisms of action in AD pathogenesis are partially unknown^5^.

Other genetic risk factors of AD and VaD still need to be found, and it seems likely that genetic variants in relation to critical biological processes constitute potential candidates^6^. Interestingly, recent evidence supports an important role of blood–brain barrier (BBB) dysfunction in both entities^7–9^. In that regard, three BBB endothelial proteins, vascular endothelial growth factor (VEGF), type 2 VEGF receptor (VEGFR2), and endothelial nitric oxide synthase (eNOS), have been proposed to underlie the onset and progression of the pathological hallmarks in AD as well as affect the response of the brain to vascular disease^10–12^.

VEGF is a cytokine induced by hypoxia that favours vascular permeability and angiogenesis, neuroprotection, neuronal survival, regeneration, differentiation and axonal outgrowth^13,14^. Increased concentrations of VEGF have been reported in cerebral vessels, neurons and reactive astrocytes in the neocortex of AD patients, and especially within Aβ plaques. Hence, continuous sequestration of VEGF into amyloid plaques during the progression of AD has been suggested to provoke deficiency of available VEGF, and therefore vascular dysfunction and neurodegeneration^18^. Additionally, high levels of VEGF have been found in cerebrospinal fluid (CSF) of patients with VaD^19^. A few studies have investigated the association between the *VEGF* gene (6p21.3) and AD with inconsistent results; whereas the haplotype GTC at G-1154A, G-7A, and C13553T of the *VEGF* gene has been associated with VaD in Koreans^20,21^.

VEGFR2 (also called kinase insert domain-containing receptor [KDR]) is a key receptor for VEGF. VEGF-VEGFR2 signalling has been involved in the development of vascular diseases, as VEGF-VEGFR2 binding promotes angiogenesis, i.e., proliferation, migration and survival of the endothelial cell^13,22^. Besides, an anti-angiogenic effect of Aβ peptides in AD have been partially attributed to the fact that Aβ1-42 is able to compete with VEGF by interacting directly with VEGFR2^23^. Despite the biological and pathological significance of VEGFR2, research concerning the role of *VEGFR2* gene (4p12) on the risk of dementias is lacking.

eNOS catalyzes the conversion of amino acid L-arginine to nitric oxide (NO) in endothelial cells where it helps maintain homeostasis by inducing vasodilatation, anti-inflammatory, antithrombotic and antiproliferative properties^24^. In AD, loss of eNOS contributes to cerebral blood vessels stiffening thereby diminishing clearance of Aβ and, in cultured human brain microvascular endothelium, increasing expression of β-amyloid protein precursor (AβPP) and β-site APP-cleaving enzyme 1 (BACE) thus favouring production of cytotoxic Aβ peptides^25–27^. eNOS also modulates synaptic function in the hippocampus, which is the first and most severely affected brain region in the pathogenesis of AD^28^. Therefore, the *NOS3* gene (7q35) has been proposed as candidate for association with VaD and AD. So far, studies in several AD populations yielded conflicting results^29^. An increased risk of incident dementia in stroke survivors older than 75 years from the UK has been reported, but this study evaluated poststroke dementia (not only VaD)^30^.

On this basis we investigated whether the *VEGF* rs699947, *VEGF* rs833061, *VEGFR2* rs2071559 and *NOS3* rs1799983 polymorphisms influence or not the susceptibility to VaD and late-onset AD, as well as disease progression, in a Spanish population.

## Results

This study included 150 VaD patients (74 large-vessel VaD and 76 small-vessel VaD), 147 AD patients, and 150 controls. Baseline characteristics of patients and controls are summarized in Table 1. Mean age at onset was 75,5 (SD 6,8) years for AD and 74,2 (SD 7,4) years for VaD. Compared with controls separately, VaD cases were well-matched in terms of gender, but were significantly younger and less educated. On the contrary, AD patients showed a higher number of females than controls (63.3 vs. 49.3, *P* = 0.016), while no differences were found in terms of age or education. As expected, VaD patients had a higher prevalence of hypertension, diabetes mellitus, hypercholesterolemia, and heavier alcohol consumption. Analyzing demographic and vascular risk factors by VaD subtype, both large-vessel and small-vessel VaD cases were younger and had higher alcohol intake history as compared with controls, albeit only small-vessel VaD patients had a significantly lower education, more hypertension, diabetes mellitus and hypercholesterolemia than controls (all *P* < 0.05). In contrast, AD participants had less hypertension and diabetes mellitus percentage compared to the control group. For this reason, SNPs analyses were calculated with an adjustment for these variables in the different groups.

**Table 1:** Baseline characteristics in Alzheimer´s disease and vascular dementia patients and controls.

Characteristics	Control N = 150	AD N = 147	*P*^a^	VaD Total sample N = 150	*P*^b^	Large-vessel VaD *n* = 74	*P*^c^	Small-vessel VaD *n* = 76)	*P*^d^
Age, years, mean (SD)	81.2 (5)	79.8 (6.9)	0.276	78.6 (7.2)	**0.000**	78.2 (7.6)	**0.001**	78.9 (6.7)	**0.004**
Sex, n(% women)	74 (49.3)	93 (63.3)	**0.016**	74 (49.3)	1.000	31 (41.9)	0.294	43 (56.6)	0.303
Education, n(%)									
≤ 8 years	21 (14)	31 (21.1)	0.083	43 (28.7)	**0.013**	18 (24.3)	0.134	25 (32.9)	**0.005**
8–13 years	52 (34.7)	59 (40.1)		36 (24)		19 (25.7)		17 (22.4)	
Elementary	65 (43.3)	52 (35.4)		59 (39.3)		28 (37.8)		31 (40.8)	
High school/college	12 (8)	4 (3.4)		12 (8)		9 (12.2)		3 (3.9)	
Hypertension, n(%)	107 (71.3)	67 (45.6)	**0.000**	127 (84.7)	**0.005**	61 (82.4)	0.071	66 (86.8)	**0.009**
Diabetes mellitus, n(%)	36 (24)	17 (11.6)	**0.005**	55 (36.7)	**0.017**	27 (36.5)	0.051	28 (36.8)	**0.043**
Hypercholesterolemia, n(%)	58 (38.7)	55 (37.4)	0.824	85 (56.7)	**0.002**	35 (47.3)	0.218	50 (65.8)	**0.000**
Alcohol, n(%)	6 (4)	11 (7.5)	0.196	26 (17.3)	**0.000**	14 (18.9)	**0.000**	12 (15.8)	**0.002**
Smoking, n(%)	37 (24.7)	24 (16.3)	0.075	52 (34.7)	0.058	27 (36.5)	0.066	25 (32.9)	0.190

Statistically significant results between patients with AD or VaD and control participants, using the Chi-square test for categorical data and the unpaired t-test for the continuous data, are indicated in bold. Abbreviations: AD, Alzheimer´s disease; SD, standard deviation; VaD, vascular dementia. Pa denotes *P* < 0.05 among controls and patients with AD; Pb denotes *P* < 0.05 among controls and patients with VaD; Pc denotes *P* < 0.05 among controls and large-vessel VaD; and Pd denotes *P* < 0.05 among controls and small-vessel VaD.

A comparison of genotype frequencies of the *VEGF* rs699947 and rs833061, *VEGFR2* rs2071559 and *NOS3* rs1799983 polymorphisms between VaD and AD patients and control group is displayed in Table 2. Genotype distributions of each polymorphism in the control samples did not deviate from those expected based on the Hardy–Weinberg equilibrium. There was strong linkage disequilibrium of the *VEGF* polymorphisms at loci -2578 (rs699947) and -460 (rs833061) in the three groups (D´ = 1.0).

**Table 2:** VEGF, VEGFR2 and NOS3 gene polymorphisms, vascular dementia and Alzheimer´s disease: association statistics.

SNP	Controls N = 150	Vascular Dementia			Alzheimer´s disease		
		Cases N = 150	OR (95%CI)	*P* value	Cases N = 147	OR (95%CI)	*P* value
*VEGF* rs699947							
CC	40 (26.7)	49 (32.7)	1.00 (Reference)		50 (34.0)	1.00 (Reference)	
CA	69 (46.0)	66 (44.0)	0.81 (0.46–1.40)	0.445	69 (47.0)	0.63 (0.35–1.13)	0.122
AA	41 (27.3)	35 (23.3)	0.70 (0.37–1.32)	0.274	28 (19.0)	**0.45 (0.22–0.91)**	**0.027**
CC vs CA + AA (dominant)			0.77 (0.46–1.28)	0.310		**0.56 (0.33–0.97)**	**0.040**
CC + CA vs AA (recessive)			1.25 (0.73–2.13)	0.415		1.68 (0.92–3.07)	0.094
HWE exact test (p)	0.327						
*VEGF* rs833061							
TT	40 (26.7)	49 (32.7)	1.00 (Reference)		50 (34.0)	1.00 (Reference)	
TC	69 (46.0)	66 (44.0)	0.81 (0.46–1.40)	0.445	69 (47.0)	0.63 (0.35–1.13)	0.122
CC	41 (27.3)	35 (23.3)	0.70 (0.37–1.32)	0.274	28 (19.0)	**0.45 (0.22–0.91)**	**0.027**
TT vs TC + CC (dominant)			0.77 (0.46–1.28)	0.310		**0.56 (0.33–0.97)**	**0.040**
TT + TC vs CC (recessive)			1.25 (0.73–2.13)	0.415		1.68 (0.92–3.07)	0.094
HWE exact test (p)	0.327						
*VEGFR2* rs2071559							
AA	38 (25.3)	37 (24.7)	1.00 (Reference)		46 (31.3)	1.00 (Reference)	
AG	82 (54.7)	63 (42.0)	0.84 (0.47–1.49)	0.556	62 (42.2)	0.63 (0.35–1.14)	0.124
GG	30 (20.0)	50 (33.3)	1.75 (0.91–3.38)	0.095	39 (26.5)	0.95 (0.47–1.92)	0.883
AA vs AG + GG (dominant)			1.09 (0.64–1.86)	0.763		0.71 (0.41–1.25)	0.236
AA + AG vs GG (recessive)			**0.51 (0.30–0.87)**	**0.014**		0.78 (0.43–1.42)	0.415
HWE exact test (p)	0.237						
*NOS3* rs1799983							
GG	65 (43.3)	56 (37.3)	1.00 (Reference)		48 (32.7)	1.00 (Reference)	
GT	63 (42.0)	70 (46.7)	1.29 (0.78–2.14)	0.320	69 (46.9)	1.56 (0.90–2.70)	0.115
TT	22 (14.7)	24 (16.0)	1.24 (0.62–2.48)	0.550	30 (20.4)	1.38 (0.66–2.87)	0.393
GG vs GT + TT (dominant)			1.75 (0.80–2.05)	0.311		1.50 (0.90–2.51)	0.124
GG + GT vs TT (recessive)			0.93 (0.49–1.76)	0.814		0.92 (0.47–1.79)	0.798
HWE exact test (p)	0.299						

OR (95%CI) and *P* values obtained with multivariate unconditional logistic regression analysis by adjusting for age and gender as covariates. Significant *P* values (< 0.05) are displayed in bold.

*CI* confidence interval, *OR* odds ratio, *SNP* single nucleotide polymorphism.

When the genetic data were analyzed adjusting for age and gender with multivariate logistic regression analysis, carrying the A allele of the *VEGFR2* rs2071559 polymorphism was found to diminish in a half the risk of developing overall VaD in the recessive model, *P* = 0.014 OR = 0.51 (0.30–0.87). None of the SNPs rs699947 and rs833061 for *VEGF* and rs1799983 for *NOS3* was related to VaD risk (Table 2).

The global study of susceptibility in AD patients showed that carriers of AA genotype and CA + AA (A-allele bearing) genotypes in the SNP *VEGF* rs699947 polymorphism as well as carriers of CC genotype and TC + CC (C-allele bearing) genotypes in the SNP *VEGF* rs833061 polymorphism had a decreased risk to develop AD, both in the codominant model, *P* = 0.027 OR = 0.45 (0.22–0.91), and the dominant model, *P* = 0.040 OR = 0.56 (0.33–0.97) after adjustment for age and gender with multivariate logistic regression analysis. No other associations were found in the rest of SNPs between AD cases and controls (Table 2).

Analysis according to the VaD subtype (small-vessel VaD or large-vessel VaD) showed an association between the GG genotype of *VEGFR2* rs2071559 and higher risk to suffer from small-vessel VaD, *P* = 0.011 in codominant model, OR = 2.91 (1.28–6.63); likewise, those with the allele A had a lower risk to develop this subtype of VaD, *P* = 0.002 in recessive model, OR = 0.37 (0.20–0.70), which persisted statistically significant after controlling for education and vascular risk factors as covariates (Table 3). Conversely, no associations were found for *VEGFR2* rs2071559 polymorphism when genotypes were compared between large-vessel VaD and control participants.

**Table 3:** VEGFR2 gene polymorphism and large-vessel and small-vessel vascular dementia subgroups: association statistics.

SNP	Large-vessel VaD					Small-vessel VaD				
	Case *n* = 74	OR (95%CI)	*P*	aOR (95%CI)	*P*a	Case *n* = 76	OR (95%CI)	*P*	aOR (95%CI)	*P*a
*VEGFR2* rs2071559										
AA	24 (32.4)	1.00 (Ref.)		1.00 (Ref.)		13 (17.1)	1.00 (Ref.)		1.00 (Ref.)	
AG	31 (41.9)	0.67 (0.34–1.33)	0.260	0.73 (0.35–1.48)	0.379	32 (42.1)	1.14 (0.53–2.43)	0.744	1.11 (0.48–2.54)	0.808
GG	19 (25.7)	1.06 (0.48–2.35)	0.889	0.99 (0.42–2.31)	0.975	31 (40.8)	**2.91 (1.28–6.63)**	**0.011**	**2.59 (1.06–6.35)**	**0.038**
Dominant		0.78 (0.41–1.47)	0.447	0.79 (0.41–1.56)	0.502		1.61 (0.79–3.28)	0.193	1.53 (0.70–3.34)	0.284
Recessive		0.74 (0.37–1.46)	0.384	0.83 (0.40–1.70)	0.611		**0.37 (0.20–0.70)**	**0.002**	**0.41 (0.21–0.82)**	**0.011**

OR (95%CI) and *P* values obtained with multivariate unconditional logistic regression analysis by adjusting for age and gender as covariates. aORs (95%CI) and Pa values adds education, hypertension, type 2 diabetes mellitus, hypercholesterolemia, smoking and alcohol consumption as covariates. Significant *P* values (< 0.05) are displayed in bold.

*aOR*,adjusted odds ratio, *CI* confidence interval, *OR* odds ratio, *ref* reference, *SNP* single nucleotide polymorphism, *VaD* vascular dementia.

Due to the great significant differences in carriers of the *APOE* ε4 allele between AD patients (40.8%) and controls (11.3%), *P* = 0.0001 OR = 5.90 (3.16–11.01), a second analysis in these groups was proposed by *APOE* rs429358 ε4 status. Among the subgroup of *APOE* ε4 non-carriers, *VEGF* rs699947 AA and rs833061 CC genotypes were significantly associated with a decreased AD risk in the codominant model, *P* = 0.021 OR = 0.38 (0.17–0.86); *P* = 0.021 and, similarly, participants with the C allele in the SNP *VEGF* rs699947 or the T allele in the SNP *VEGF* rs833061 had higher risk to suffer from AD in our sample, *P* = 0.033 in recessive model, OR = 2.17 (1.06–4.42), which remained statistically significant after controlling for vascular risk factors. Among the subgroup of *APOE* ε4 carriers, although not statistically significant a tendency in *VEGF* rs699947 and rs833061 was found, that reached significant differences under the heterozygous additive and codominant models (*P* = 0.026) after correction for education and vascular risk factors as covariates (Table 4).

**Table 4:** VEGF gene polymorphism and Alzheimer´s disease by APOE ε4 status: association statistics.

*VEGF* rs699947	Controls N = 150	Alzheimer Disease				
Characters		Cases N = 147	aOR (95% CI)^1^	*P* value^1^	aOR (95% CI)^2^	*P* value^2^
*APOE ε4(* +*)*	n = 17	n = 60				
CC	3 (17.6)	18 (30.0)	1.00 (Reference)		1.00 (Reference)	
CA	11 (64.8)	27 (45.0)	0.21 (0.04–1.02)	0.053	**0.13 (0.02–0.78)**	**0.026**
AA	3 (17.6)	15 (25.0)	0.31 (0.04–2.26)	0.247	0.19 (0.02–1.68)	0.135
Dominant			0.23 (0.05–1.05)	0.059	**0.14 (0.02–0.79)**	**0.026**
Recessive			1.18 (0.23–5.95)	0.845	1.43 (0.25–8.09)	0.688
APOE ε4(-)	n = 133	n = 87				
CC	37 (27.8)	32 (36.8)	1.00 (Reference)		1.00 (Reference)	
CA	58 (43.6)	42 (48.3)	0.75 (0.39–1.42)	0.374	0.73 (0.37–1.45)	0.372
AA	38 (28.6)	13 (14.9)	**0.38 (0.17–0.86)**	**0.021**	**0.36 (0.15–0.85)**	**0.019**
Dominant			0.61 (0.33–1.11)	0.104	0.59 (0.31–1.11)	0.100
Recessive			**2.17 (1.06–4.42)**	**0.033**	**2.25 (1.07–4.77)**	**0.033**

1Adjusted ORs (95%CI) and *P* values obtained with multivariate unconditional logistic regression analysis by adjusting for age and gender as covariates. 2Adjusted ORs (95%CI) and *P* values of all models on the basis of risk factors such as age, gender, education, hypertension, type 2 diabetes mellitus, hypercholesterolemia, smoking and alcohol consumption. Significant *P* values (< 0.05) are displayed in bold.

*aOR* adjusted odds ratio, *CI* confidence interval.

**Figure 1:**
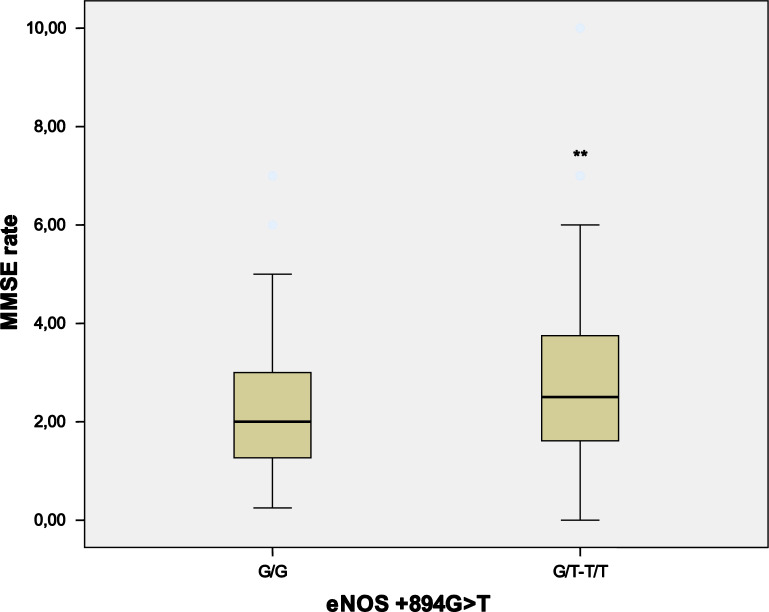
NOS3 (also so-called eNOS) rs1799983NOS G/G genotype determines a less rapid cognitive decline in Alzheimer´s disease. The study included 147 (GG:48; GT:69; TT:30) patients with AD. Scores of the MMSE rate (MMSE decay/follow-up time expressed in years) derived from GG and GT + TT genotypes were measured. Box plots show median values (horizontal line inside the box), quartiles (box boundaries), and the largest and smallest observed values (error bars). Statistical significance for difference using Mann–Whitney U test: **P < 0.05 compared with GG genotype.

We next investigated the role of SNPs in *APOE*, *VEGF*, *VEGFR2* and *NOS3* genes on disease progression. The *NOS3* rs1799983 GG genotype was found to be an independent protective factor of rapid progression, *P* = 0.048 OR = 0.22 (0.05–0.99) after adjusting for classic factors usually affecting AD progression (Table 5). The MMSE rate (MMSE decay/follow-up time expressed in years) values were also significantly lower in *NOS3* rs1799983 GG when compared with GT + TT genotypes (*P* = 0.035) (Fig. 1). Further a general lineal model confirmed that *NOS3* rs1799983 T allele was an independent marker for faster decline, *P* = 0.028, B 0.556 (0.061–1.051), after covariate adjustment. However, for *APOE*, *VEGF* and *VEGFR2* genotypes, the lack of association was the rule among analyzed end points (Table 5).

**Table 5:** Logistic regression analysis showing independent variables associated with rapid progression of Alzheimer´s disease.

Variable	Rapid progressors		
	OR	95% CI	*P* value
Age at diagnosis	0.93	0.86–1.02	0.130
Sex	0.36	0.11–1.25	0.107
Education	0.65	0.31–1.33	0.240
hypertension	**0.24**	**0.06–0.99**	**0.049**
type 2 DM	1.61	0.20–12.83	0.652
hypercholesterolemia	0.98	0.23–4.14	0.978
smoking	0.27	0.04–1.75	0.169
alcohol	1.09	0.10–11.48	0.942
Depression	1.29	0.41–4.02	0.665
Psychosis	**5.50**	**1.44–20.98**	**0.013**
AChEI/memantine	0.82	0.45–1.47	0.502
Follow-up time	**0.44**	**0.29–0.65**	**0.0001**
*APOE* ε4 carriers	1.24	0.36–4.26	0.733
*VEGFA* TT vs TC-CC	1.77	0.52–6.07	0.364
*VEGFR2* AA vs AG-GG	2.98	0.86–10.37	0.086
*NOS3* GG vs GT-TT	**0.22**	**0.05–0.99**	**0.048**

OR and their 95% CI were calculated to demonstrate the independent association between good prognosis and eNOS GG genotype. Significant *P* values (< 0.05) are displayed in bold.

*AChEI* cholinesterase inhibitor, *CI* confidence interval, *OR* odds ratio.

## Discussion

We conducted a case–control study to investigate the relationship between several endothelial function-related gene polymorphisms, VaD and AD in a Spanish population. At the epidemiological level, there were more women in the group of participants with AD than in the control group, in agreement with several studies in Europe. The higher prevalence of AD in women could be in some extent explained due to differences in the following factors: a) longevity and survival bias -there are more women at older ages, when the development of AD is more likely-, b) comorbidities -e.g., women have twice the risk of depression at midlife, which is believed to increase the risk of AD-, c) biological hormonal factors -indeed, oophorectomy, menopause and androgen-deprivation therapy have been associated with deleterious cognitive changes in the literature-; and d) sociocultural factors -in the past century, women had fewer opportunities for higher education and occupational attainment and also exercise less than men at midlife, and both education and exercise are associated with a decreased risk of AD^31^.

The major findings in our study are that *VEGFR2* rs2071559 A allele protects against VaD, whereas *VEGF* rs699947 A and rs833061 C alleles show to decrease the risk of AD and *NOS3* rs1799983 influences disease progression. Our data also show strong linkage disequilibrium between the SNPs in *VEGF* gene analyzed (rs699947 A and rs833061 C), which is consistent with prior studies^32^.

Noteworthy, when we grouped the patients by VaD subtype, the *VEGFR2* -604A allele conferred a significant decreased risk for small-vessel VaD, but not for large-vessel VaD, suggesting a difference in the pathophysiological mechanisms underlying both VaD subtypes. Although there have been no prior reports of an association between *VEGFR2* polymorphisms and VaD in any population, these results are in agreement with existing literature in stroke patients, which is limited to Asian population. Oh et al. observed that individuals carrying the *VEGFR2* + 1719 T allele had an increased risk of ischemic stroke in small-vessel disease (SVD) patients. Despite the fact that there was no association between SNP − 604 and SNP + 1192 and ischemic stroke risk, GGT, GAT and GGT haplotypes of -604A > G, + 1192G > A, and + 1719A > T *VEGFR2* polymorphisms increased risk of ischemic stroke^33^. In another Chinese study, the + 1192A allele was associated not only with increased susceptibility to intracerebral haemorrhage (ICH), but also it was a prognostic factor for stroke recurrence, whereas the -604G allele predicted a reduced susceptibility to atherothrombotic stroke and stroke recurrence, and was reversely correlated with carotid artery intima media thickness^34^. Thereafter, Han et al. described a higher risk of silent brain infarcts in men and younger than 65 years carrying the -604G allele in a Korean population^35^.

VEGFR2 is the main receptor for VEGF in endothelial cells and endothelial progenitor cells. Binding of VEGF to VEGFR2 is followed by activation of downstream signalling pathways such as PI3-K/Akt, PLC/PKC, Src, MEK/ERK and eNOS, which are essential for migration, proliferation and survival of endothelial cells, thereby stimulating angiogenesis^14,36^. VEGF can also prevent oxidized-LDL–induced endothelial cell damage via an intracellular glutathione-dependent mechanism through VEGFR2^37^. Several SNPs of *VEGR2* are known to inhibit the activity of VEGF-VEGFR2 signalling pathway. The minor G allele of SNP –604A > G leads to structural alteration of the binding site for transcriptional factor E2F (involved in cell cycle regulation, interacting with Rb p107 protein) in VEGFR2 gene promoter region, which suppresses VEGFR2 expression by 68%. The minor A allele of SNP + 1192G > A in exon 7 and the minor T allele of SNP + 1719A > T in exon 11 have been found to reduce binding affinity of VEGF to VEGFR2^32,38^.

Interestingly, the different consequences for different strokes subtypes of genetic variants in *VEGFR2* gene could be explained by a dual role of the VEGF-VEGFR2 system, enhancing both physiological and pathological angiogenesis^35^. It has been proposed that the allele -604G in *VEGFR2* gene, by down-regulating VEGF-VEGFR2 signalling, causes disrupted endothelial cell development and defective blood vessel formation, decreases the integrity of vascular endothelium as well as inhibits endothelial repair, eventually leading to small-arterial occlusion and SVD^34,35,39^. Similarly, vascular degeneration and formation of weak, thin-walled vasculature can reduce vessel compliance and increase the risk of spontaneous vessel wall rupture and ICH under some stresses such as hypertension and increased shear stress^34,40^. In contrast, the allele -604G could exert a protective effect on large-artery atherosclerosis by reducing neovascularisation and inflammation, retarding atherosclerotic lesions growth and the plaque destabilization leading to rupture in major cranial arteries^34,35^. Albeit the decrease in VEGFR2 function could inhibit atherosclerosis, a damaging effect diminishing the maintenance of endothelial integrity may be more profound^35^. Noteworthy, though the degree of VEGFR2 expression in small artery myocytes has been associated with the aging brain, the relationship between VEGFR2 and small-vessel VaD in our research is preserved irrespective of age^41^.

Besides its involvement in the vascular system, neurotrophic effects of VEGF have also been ascribed to VEGFR2 signal^36^. In ischemic stroke, VEGF is induced in the ischemic border zone and acts on local neurons to promote neuroprotection^42^. VEGF also stimulates neurogenesis in the subventricular zone of the lateral ventricles and in the subgranular zone of the hippocampal dentate gyrus, from which new neurons migrate to the site of ischemia^43^. Hence, functional recovery following stroke depends partly on neuronal plasticity in non-ischemic regions^44^. In this regard, one study evidenced that the beneficial effects of bone marrow mononuclear cells inoculation in an animal model of VaD (which increase level of VEGF as well as levels of p-Raf1 and p-ERK –downstream proteins in the VEGFR2 signalling pathway–, increase vascular density, reduce white matter lesions, and finally, lead to a better cognitive outcome) were abolished by using VEGFR2 inhibitor SU5416^45^.

On the other hand, carriers of the *VEGF* -2578A and -460C alleles, both SNPs in linkage disequilibrium in the promoter region of the gene, decreased the risk to suffer from AD in our study. In vitro experiments have demonstrated that VEGF binding to Aβ-40 and Aβ-42 within the amyloid plaques in the brain of AD patients might result in sequestration and local deficiency of available VEGF and, subsequently, contribute to insufficient vascularisation and reduced cerebral perfusion^16–18^. Cerebral hypoperfusion is common in AD, initially in the posterior cingulate and precuneus areas, and later in medial temporal regions^46^. VEGF elevations have been postulated to counteract these deleterious effects of the AD pathological cascade by enhancing vascular survival^47^. In fact, patients with AD exhibited lower levels of cerebral capillary VEGF expression in the hippocampus, superior temporal cortex, and brainstem than controls, whereas treating AD mice models with cells secreting VEGF yielded reductions in memory impairment, tau and amyloid burden^48–50^. In addition, Hohman et al. reported that increased levels of VEGF in CSF were associated with improved hippocampal volume, episodic memory, and executive function^51^.

Nevertheless, CSF levels of VEGF in patients with AD are discordant. Tarkowski et al. evidenced higher CSF levels of VEGF in AD and VaD than controls; Blasko et al. demonstrated no difference in CSF VEGF levels between AD and controls; and data from the Alzheimer’s Disease Neuroimaging Initiative (ADNI) found lower CSF levels of VEGF capable to distinguish AD from controls with 76% sensitivity and a 84% specificity^19,52,53^. This inconsistence agrees with the results from a meta-analysis containing 7 studies (2731 AD patients and 2442 controls), in which 3 studies observed an association between *VEGF*-2578C > A polymorphisms and risk of AD and 4 studies did not^20^. Similarly, contradictory results have also been reported about serum VEGF levels in AD patients, independently of VEGF genotypes^18,54^. We consider some concurrent diseases that might up-regulate VEGF (e.g., tumours, ischemia, trauma and inflammation), differences in technologies used, sample sizes and populations included would explain variations in CSF and serum levels of VEGF^55^. We excluded participants with these disorders mentioned above in our work in order to avoid confusion factors.

Finally, we demonstrated that a rapid decline occurs in patients with AD if the *NOS3* rs1799983 T allele is present. This category of patients consistently seems to have a more aggressive disease and thus needs particular attention at follow-up. It has been suggested that the 894G > T polymorphism in the *NOS3* gene influences AD development by increasing the production of endothelial NO^56,57^. In AD brains, the deposits of Aβ can generate superoxide radicals that react with NO to form peroxynitrate, which can cause oxidative stress to further accelerate neurodegenerative changes leading to AD^26–28,58–60^. In this sense, Chrysohoou et al. observed that compared with the *NOS3* 894GG genotype, carriers of the 894TT genotype had higher levels of inflammatory and oxidative stress markers including fibrinogen, leukocytes, oxidized low-density lipoprotein cholesterol, homocysteine, C-reactive protein and Aβ levels, all of them often observed in neurodegenerative processes^61^.

Although *APOE* is well characterized as a disease risk modulator, its importance as a predictor of progression is not confirmed in the present study, which supports the results of a meta-analysis suggesting that the presence of the *APOE* ε4 allele does not contribute to the rate of cognitive decline in persons with AD^62^. The strength of our analysis is that we kept in mind many factors that have been related to disease progression (i.e., education, psychotic symptoms, treatment with any cholinesterase inhibitor, memantine, or both) and could distort observed associations^63^.

The main limitation of this study is that it is only of moderate size. Therefore, future studies with a larger subject size would be necessary to examine the potential importance of *VEGFR2* and *VEGF* genes as novel genetic risk markers for VaD (particularly small-vessel VaD) and AD, respectively, in addition to the influence of *NOS3* gene on AD progression. Our findings would also need to be validated in other ethnic groups.

In conclusion, our results provide evidence of the putative role of some polymorphisms in endothelial function-related genes as genetic susceptibility factors in both VaD and AD and boost to further investigate angiogenesis as a new target for dementia prevention and treatment.

## Materials and methods

### Study design and population

Patients enrolled in this case–control study (160 VaD patients and 160 AD patients) were recruited consecutively from September 2005 to January 2007 within the Neurology Department, Complejo Asistencial Universitario de Salamanca [CAUSA] (Salamanca, Spain) and from March 2011 to January 2012 within the Neurology Division, Complejo Asistencial de Ávila (Avila, Spain) and Outpatients Departments from which we receive referrals. The inclusion of AD patients started in September 2011. Neuropsychological protocols, a detailed structured interview, and clinical examinations were performed. All patients had morphologic and/or functional neuroradiological testing together with the usual battery of screening blood tests to exclude treatable causes of dementia. The *National Institute on Aging and Alzheimer's Association* (NIA-AA) criteria were fulfilled by patients with AD^64^, and the *National Institute of Neurological Disorders and Stroke and the Association Internationale pour* *la Recherche* *et l’Enseignement en Neurosciences* (NINDS-AIREN) criteria by patients with VaD^65^. In addition, VaD patients were classified according to the radiological NINDS-AIREN criteria as having large-vessel or cortical VaD [cVaD] (strategic large-vessel infarct of the dominant hemisphere or bilateral hemispheric strokes) or small-vessel or subcortical VaD [sVaD] (white-matter hyperintensities involving at least a quarter of the white matter, multiple lacunes or bilateral thalamic lesions)^65^. We exclude patients with ischemic-hypoperfusive or hemorrhagic VaD, as well as those presenting both vascular and Alzheimer features (mixed dementia). All dementia cases were defined as sporadic VaD or late-onset AD because there was neither an autosomal dominant dementia trait nor a first degree relative diagnosed with familial dementia. Cognitive impairment was assessed using the Mini-Mental State Examination (MMSE)^66^. Cognitively healthy controls (n = 160) were recruited consecutively from individuals older than 75 years who attended a health screening in the outpatient clinics of the participating institutions, from June 2011 to November 2011. Assessment of controls included a full medical history and a physical examination. All the healthy controls that had MMSE scores of ≤ 28 and a history of neurological or psychiatric disease were excluded. Furthermore, none of the patients or controls was affected by cancer or chronic inflammatory diseases.

All the individuals included in the study were of Caucasian origin and live in Castilla y Leon, a central-western region of Spain. The study protocol was in accordance with the Declaration of Helsinki, approved by the clinical research ethics committees of the healthcare areas of Salamanca and Avila and complied with Spanish data protection law (LO 15/1999) and specifications (RD 1720/2007). Written informed consent was obtained from the patients or their legal guardians when patients had serious cognitive impairment and control participants. For each participant, a questionnaire was administered to gather information on demography, vascular risk factors and life style. The following variables were included in the analyses: a history of hypertension (blood pressure ≥ 140/90 mmHg measured on the right arm in supine position at two different occasions in an interval of at least two weeks in between or diagnosis of hypertension previous to the use of anti-hypertensive medication), type 2 diabetes mellitus (symptoms with random glucose > 200 mg/dL [11,1 mmol/L], fasting plasma glucose > 126mg/dL [7 mmol/L], plasma glucose two hours after a 75 g oral glucose tolerance test > 200 mg/dL [11,1 mmol/L] or diagnosis of diabetes previous to the use of diabetic medication), hypercholesterolemia (total cholesterol > 190 mg/dL or LDL-cholesterol > 115 mg/dL, or previous diagnosis and cholesterol lowering diet or drugs), tobacco use (any current smoking status or ex-smokers for less than 5 years), and alcohol consumption (alcohol intake ≥ 40 g per day). We collected a blood sample from each participant in a tube containing sodium EDTA.

Moreover, 152 AD patients from the AD group underwent neurological evaluation by a neurologist at least in three different evaluations (basal plus two follow-up examinations). For the disease progression calculation, we used MMSE variation during follow-up. More precisely, for each patient, we computed MMSE values and their timing of administration, for example, at the time of AD diagnosis (basal) and at the last available follow-up data point for MMSE scale. AD patients were classified according to the cut-off established by Cortes et al.^67^ as having rapid progression (individuals with MMSE rate, MMSE decay/follow-up time expressed in years, higher than 4.5) or normal progression (AD patients with MMSE score point decrease per year lower or equal to 4.5).

After further exclusion of participants without blood samples and patients without clinical follow-up data available, a total of 150 VaD patients (sVaD-74, cVaD-76) 147 AD patients and 150 controls were included for data analysis (response rate: 93.8% for VaD and controls and 91.9% for AD).

### DNA isolation and genotyping

DNA was extracted from peripheral blood leukocytes following the phenol–chloroform method. Determination of the single nucleotide polymorphisms (SNPs) of + 334 T > C (E4; rs429358) in apolipoprotein E (APOE), -2578C > A (rs699947) and -460C > T (rs833061) in the promoter region of VEGF, -604A > G (rs2071559) in the promoter region of VEGFR2 and + 894G > T (rs1799983) in exon seven of eNOS were carried out with TaqMan_ SNP Genotyping Assays (assay ID: c_3084793_20, c_1647381_10, c_8311602_10, c_158969271_10 and C_3219460_20, respectively) on a ABI Prism 7300 HT Real-Time polymerase chain reaction (RT-PCR) System (Applied Biosystems Inc., Foster City, CA). The reactions were set up in a 96-well plate and were performed in a final volume of 10 μl with 0.5 μl of sample DNA, 0.25 μl TaqMan-specific assay, 4.25 μl distilled H_2_0 and 5 μl Taq-Man Genotyping Master Mix. The amplification conditions were as follows: 60 ºC initial denaturation for 30 s followed by 40 cycles of 95 ºC denaturation for 10 min, 95 ºC annealing for 15 s and elongation at 60 ºC for 1 min. In all PCR reactions, a final elongation step was applied at 60 ºC for 30 s. To ensure the reproducibility, a 5% of random samples were re-genotyping.

### Statistical analysis

In baseline characteristics, categorical variables − presented as numbers and percentages − were analyzed by using the χ2 test, whereas the Student´s *t* test was required for continuous variables − expressed as mean values ± standard deviation (SD) − when the normality of the distribution was determined by the Kolmogorov–Smirnov test. The χ2 test was also used to assess the Hardy–Weinberg equilibrium was accomplished in control group participants for each polymorphism and to compare genotype frequencies between cases and controls. The relative risk of dementia for each polymorphism was estimated in odds ratios (ORs) and 95% confidence intervals (CIs). Three genetic models were taken into account: the dominant model (Mm + mm vs. MM), the codominant model (mm vs. Mm vs. MM), and the recessive model (mm vs. MM + Mm); in which “M” indicates the major allele and “m” the minor allele. Associations of SNPs with VaD and AD were analyzed by multivariate logistic regression adjusted for possible confounders, including age, gender, education, *APOE ε4* allele and vascular risk factors. We applied the Bonferroni´s correction for multiple comparisons since some stratification of the samples was performed.

Three different measurements were employed to assess the influence of SNPs on disease progression, in order to determine consistent effects regardless of statistical method or covariates selected. The Mann–Whitney U test and a general lineal model were used to evaluate the role of each SNP in MMSE rate (MMSE decay/follow-up time [in years]), whereas logistic regression analysis, after adjusting for factors generally affecting AD progression^68^ (age at diagnosis, gender, education, depression, psychosis, treatment with cholinesterase inhibitors, memantine, or both, and follow-up time), was calculated to investigate genotype effects on disease progression phenotypes. . Depression and psychosis (delusions/hallucinations) were assessed at an interview with a responsible caregiver by using the corresponding sub-scales in the Neuropsychiatric Inventory^69^. Each sub-scale has an entry question inquiring whether the disturbance had been present in the last month. If the answer is affirmative, the caregiver is asked to rate the specific symptom on a four-point frequency and on a three-point severity scale; subsequently, frequency and severity scores are multiplied (composite score). A composite score of 4 or more on an individual subscale (delusions or hallucinations) or 6 or more on a combined subscale (delusions plus hallucinations) was used to identify the presence of psychosis clinically relevant, whereas a composite score of 4 or more on the depression domain was required for a diagnosis of depression.

Statistics were carried out with the statistical software package SPSS (version 21.0. SPSS Inc. [Chicago, IL]). For all statistical analyses, *P-*values < 0.05 were considered to reach statistical significance.

## Data Availability

The datasets generated during and/or analysed during the current study are available from the corresponding author on reasonable request.
